# Efficacy of an environmental enrichment intervention for endometriosis: a pilot study

**DOI:** 10.3389/fpsyg.2023.1225790

**Published:** 2023-10-10

**Authors:** Grace De Hoyos, Darlenne Ramos-Sostre, Annelyn Torres-Reverón, Bárbara Barros-Cartagena, Verónica López-Rodríguez, Cristina Nieves-Vázquez, Fanny Santiago-Saavedra, Caroline B. Appleyard, Eida M. Castro, Idhaliz Flores

**Affiliations:** ^1^School of Behavioral and Brain Sciences, Ponce Health Sciences University, Ponce, Puerto Rico; ^2^Department of Basic Sciences, Ponce Health Sciences University, Ponce, Puerto Rico; ^3^Sur180 Therapeutics, Inc., McAllen, TX, United States; ^4^Department of Obstetrics and Gynecology, Ponce Health Sciences University, Ponce, Puerto Rico

**Keywords:** endometriosis, randomized clinical trial, pelvic pain, anxiety, depression, quality of life, stress, environmental enrichment

## Abstract

**Introduction:**

We have previously shown that Environmental Enrichment (EE), a multi-modal psychosocial intervention consisting of increased social interaction, novelty, and open spaces, improved disease presentation, anxiety, and immune-related disturbances in the rat model of endometriosis. However, there is a knowledge gap regarding the effects of EE interventions in patients with this painful, inflammatory chronic disease.

**Aim:**

To adapt and test the efficacy of an EE intervention on pelvic pain, mental health, perceived stress, quality of life, and systemic inflammation in endometriosis patients through a randomized clinical trial (RCT).

**Materials and methods:**

A multidisciplinary team with expertise in physiology, neuroscience, psychology, and women’s health adapted and implemented a two-arm RCT comparing an EE intervention with a wait-list control group. Six EE modules administered on alternate weeks were provided to patients in the intervention (*N* = 29); controls received education only. Survey data and biospecimens were collected at baseline, end-of-study, and 3-months post-intervention to assess pain (Brief Pain Inventory, BPI), endometriosis-related quality of life-QoL (Endometriosis Health Profile-30, EHP30), anxiety (Generalized Anxiety Disorder 7, GAD7), depression (Patient Health Questionnaire for Depression 8, PHQ8), pain catastrophizing (Pain Catastrophizing Score, PCS), stress (Perceived Stress Scale-14, PSS14), and saliva cortisol levels (AM, PM).

**Results:**

Compared to the wait-list controls, participants in the EE intervention showed significantly decreased GAD-7 scores at the end of the intervention and 3-month follow-up. Depression, perceived stress, and QoL improved at the 3-month follow-up compared to baseline. While pain levels did not improve, they significantly correlated with anxiety, depression, QoL and pain catastrophizing scores.

**Conclusion:**

This pilot RCT demonstrated significant improvements in anxiety and depressive symptoms, QoL, and perceived stress, supporting enriched environments as an integrative psychosocial intervention to be used as adjuvant to the standard of care for endometriosis pain.

## Introduction

Environmental enrichment (EE) refers to a psychosocial mind–body intervention consisting of a combination of social and cognitive stimulations (social support, novelty, and exposure to open spaces; Queen et al., 2020; Rojas-Carvajal et al., 2022). Human and animal studies have shown that EE enhances memory and cognition and alleviates symptomatology of neurological and visceral/inflammatory painful conditions (Gabriel et al., 2010; Gonçalves et al., 2018; de la Tremblaye et al., 2019; Rosbergen et al., 2019; Wang et al., 2019; Qin et al., 2021; Tapias et al., 2022; Neves et al., 2023). Moreover, EE has been shown to be effective for chronic stress and mood disorders (depression and anxiety) in both humans and animal models (Benaroya-Milshtein et al., 2004; Seong et al., 2018; Scarola et al., 2019; Wang et al., 2019; Fournier et al., 2020; Kimura et al., 2022). Recently, an indicator of active involvement in EE that measured cognitive, social, and physical activity was able to identify individuals with major depression (Flores-Ramos et al., 2022). The underlying mechanisms of the beneficial effects of EE could include regulation of immune/inflammatory factors and brain pathways related to the hypothalamus-pituitary–adrenal (HPA) axis, mood regulation, and chronic pain [(i.e., increased expression of brain-derived neurotrophic factor (BDNF) and corticotropin-releasing factor receptor type 1 (CRFR1); Sztainberg et al., 2010; Simpson and Kelly, 2011; Singhal et al., 2014, 2019; Dandi et al., 2018; Barros et al., 2019; Scarola et al., 2019; de Sousa Fernandes et al., 2022; Liew et al., 2022]. Despite the growing evidence for the effectiveness of EE interventions in animal and human studies, research on integrative interventions incorporating EE features in humans is still limited, with very few studies conducted on its efficacy for pain, stress-related, and inflammatory conditions.

It is widely accepted that the debilitating and painful symptoms of endometriosis—severe dysmenorrhea, dyspareunia, and chronic pelvic pain—cause chronic stress, psychological distress, and low quality of life (QoL) at a higher degree than in other chronic pelvic pain conditions (Centini et al., 2013; Brasil et al., 2020). The chronicity, unpredictability, and refractory nature of endometriosis symptoms negatively impact psychological health, social functioning, educational goals, work productivity and professional choices, and intimate relationships, all contributing to the detriment of the life-course potential of affected individuals (Fourquet et al., 2010, 2011; Vannuccini et al., 2018; Missmer et al., 2021). The impact of stress on disease progression, inflammation, pain perception, and symptom exacerbation has been systematically shown in animal and human studies (Cuevas et al., 2012; Hernandez et al., 2017; Cuevas et al., 2018; Casalechi et al., 2021). Moreover, controlling stress reverses disease progression (number and size of vesicles developed, inflammation) in a rodent model of endometriosis (Appleyard et al., 2015), while also improving anxiety behaviors, possibly through controlling inflammation and resetting HPA axis dysregulation. This was also demonstrated with an EE intervention (Torres-Reverón et al., 2018). Several studies have shown dysregulations in the HPA axis in endometriosis patients and correlations between cortisol levels and symptoms (Lima et al., 2006; Petrelluzzi et al., 2008; Quiñones et al., 2015). This line of research supports the notion that stress control as a therapeutic intervention could improve endometriosis symptomatology and its impact on emotional wellbeing, mental health, and QoL (Appleyard et al., 2020). However, only a few randomized clinical trials (RCTs) of psychosocial interventions aimed at stress management (i.e., yoga, psychotherapy, relaxation training) have been conducted in endometriosis patients (reviewed by Evans et al., 2019). The promising positive results regarding pain, anxiety, depression, stress, and fatigue prompted a call for conducting more well-designed RCTs including active control groups for endometriosis.

In response to this gap, we adapted and tested in patients with endometriosis a multi-level integrative intervention based on the EE paradigm shown to be effective in a rat model of the disease. Using an integrative, patient-centered methodology, we developed a psychosocial intervention consisting of activities mimicking and integrating the three hallmarks of EE: social support, novelty, and open spaces (Nieves-Vazquez et al., 2022). We hypothesized that EE would be effective in decreasing pelvic pain symptoms and improving QoL (1^ry^-outcomes), as well as improving depression and anxiety symptoms, perceived stress, and cortisol reactivity (2^ry^-outcomes). Our rigorous RCT demonstrates that EE promotes significant and long-lasting improvements in anxiety, despite the lack of benefit for pain relief. Long-term, this modality shows promising benefits for improving depression, QoL and stress. Together, these data support integrating psychosocial interventions in the clinical management of endometriosis patients.

## Methods

### Translation of the EE intervention

The process of translating the environmental enrichment (EE) paradigm has been described in detail by Nieves-Vazquez et al. (2022).

### Feasibility and acceptability of the EE intervention

The pilot trial’s acceptability and feasibility were determined by rates of Recruitment, Enrollment, Adherence, Survey completion and Module evaluations. The methods and results for these analyses have been described in detail by Nieves-Vazquez et al. (2022).

### Recruitment

After IRB approval (Protocol #1901004205), a recruitment campaign was conducted using social media (Facebook, Instagram, Twitter) of the *Fundación Puertorriqueña de Pacientes con Endometriosis* (ENDOPR). Participants were 18–50 years old with a surgical diagnosis of endometriosis, symptomatic (reporting pelvic pain of five or more on the numerical rating scale-NRS), and able to provide written informed consent. Patients with concomitant painful/inflammatory conditions or under psychiatric treatment were excluded. Additional details of the inclusion and exclusion criteria are described by Nieves-Vazquez et al. (2022).

### Study design of the pilot RCT

To evaluate the efficacy of a translated EE intervention for endometriosis patients, we conducted an RCT of parallel design with an intervention group (EE intervention; *n* = 29) and a wait-list control group (*n* = 27) from August 2021 to July 2022. Participants randomized to the intervention participated in six EE modules on alternate Saturday mornings. They could receive (or continue receiving) standard gynecological care (hormonal, analgesics, or surgeries) and psychological therapy as needed. Participants randomized to the wait-list control condition were invited to participate in an online seminar about endometriosis and could also receive standard of care for endometriosis and mental health. After providing informed consent, all subjects completed validated surveys at baseline and end of the intervention to assess QoL, perceived stress, anxiety, depression, and pain symptoms, and provided saliva samples. Individuals in the intervention group were followed up 3-months after the study ended to assess possible long-term effects of the intervention. Participants in the control group completed the same questionnaires and provided the saliva samples during house visits within 1 week before and 1 week after the timeline of the intervention. Clinical and socio-demographic data, as well as pain catastrophizing scores were obtained with the Endometriosis Phenome Project (EPHect) questionnaire (Vitonis et al., 2014). Participants in the intervention group used WhatsApp chats to continue communicating between meetings. Data regarding treatments and doctor’s visits during the study were obtained from all participants with a clinical history questionnaire administered twice during the study period.

### Study surveys

Participants in the EE condition completed the following surveys at baseline and end of the intervention, and at 3 months after the intervention was completed. Individuals in the control group completed the same questionnaires only at baseline and end of the study period:

#### Endometriosis phenome project clinical minimal questionnaire

The validated Spanish version of the EPhect’s EPQ-M survey was used to collect cross-sectional, self-reported data. Developed by the World Endometriosis Research Foundation (WERF), this survey standardizes data collection from patients, including demographics, medical history, ob-gyn history, and lifestyle (Vitonis et al., 2014). Different types of pelvic pain (dysmenorrhea, dyspareunia, chronic pelvic pain) are measured using a numerical rating scale from 1 (no pain) to 10 (worse pain; Zonta et al., 2009).

#### Endometriosis health profile-30

This survey measures the endometriosis-related health status through 30 items covering five disease-related scales (core questionnaire): pain, control and helplessness, emotional wellbeing, social support, and self-image. EHP-30 has been shown to be sensitive to changes in patient outcomes, making it a useful tool in endometriosis clinical trials. Response categories are rated on a five-point Likert scale (0 to 4). The global QoL score is converted on a scale of 0 to 100, with the lower score representing a better quality of life (Jones et al., 2004; Jenkinson et al., 2008).

#### Brief pain inventory

This questionnaire measures pain intensity (minimum, maximum, average, current) using a numerical rating scale (NRS) of 0 to 10, with the highest number representing the worst imaginable pain. This instrument also measures the degree of pain related function impairment regarding general activity, mood, walking, work (including housework and paid work), relationships, sleep, and enjoyment of life. A global pain impact score can be calculated, with higher scores indicating worse impact (Miettinen et al., 2019).

#### Perceived stress scale

This self-assessment tool measures the level of perceived stress due to life situations over the past month. It assesses to what extent respondents feel in control of unpredictable or unexpected situations, or conversely, whether they feel out of control and experience stress that leads to discomfort. It consists of 14 items with a response format of a five-point scale (0 = never, 1 = almost never, 2 = occasionally, 3 = often, 4 = very often). A high total score corresponds to a high level of perceived stress (Perera et al., 2017).

#### Anxiety general anxiety disorder-7

This self-administered questionnaire measures anxiety symptomatology through seven items which are scored from 0 to 3. The cut points of 5, 10, and 15 represent mild, moderate, and severe, respectively (Spitzer et al., 2006). Patients reporting moderate–severe scores in GAD-7 were provided with a referral for psychological services.

#### Patient health questionnaire-8

This self-administered questionnaire measures depression symptomatology through eight items which are scored from zero to three. Scores of 5, 10, 15, and 20 represent cut points for mild, moderate, moderately severe, and severe, respectively (Beck and Beamesderfer, 1974). Patients reporting moderate–severe scores in PHQ-8 were provided with a referral for psychological services.

#### Pain catastrophizing scale

This scale is part of the EPhect-Q Questionnaire that was completed at baseline. The PCS evaluates three dimensions of catastrophizing: helplessness, rumination, and magnification (Petrini and Arendt-Nielsen, 2020). The scale has a total score of 52, with items scored from 0 (“not at all”) to 4 (“all the time”; Sullivan, 2009). A PCS score higher than 30 is clinically significant and identifies those with a higher risk of chronicity and disability due to pain (Kristiansen et al., 2014).

### Saliva sampling and cortisol ELISA

Saliva samples for cortisol were collected at baseline, at the end of the intervention, and at the 3-month follow-up for the experimental group. Saliva samples (approximately 1–3 ml) were obtained by passive drool into pre-labeled 15 ml tubes. These samples were obtained at the same time of the day (between 8:00–9:00 am and at noon, before and after the intervention) to account for circadian variations in cortisol levels. For the control group, samples were collected 1 week before the start of the intervention and 1 week after during house visits. Control individuals were asked to collect saliva at the same time as the intervention group, on the same day, and store them at 4°C until collection during visits by the research team. Saliva samples were stored short-term on wet ice during transport to the research laboratory where they were processed. Once in the lab, the saliva samples were spun down to clear buccal cells, and aliquots stored at-80°C until analysis by ELISA. Cortisol analysis was done using the High Sensitivity Salivary Cortisol Enzyme Immunoassay kit (Salimetrics, State College, PA, cat #130025), validated for the quantitative measurement of salivary cortisol, following the manufacturer’s protocol. All samples were assayed undiluted in duplicate on the same assay plate. Cortisol concentrations were calculated based on standard curve, averaged, and reported in μg/dl.

### Statistical analysis

Equivalence of baseline characteristics, as well as between-group differences in clinical-demographics variables and study outcomes, were assessed using descriptive and univariate statistics, including T-test or Mann–Whitney test for continuous variables (depending on normality of distribution, as assessed by Shapiro–Wilk test), and Chi-square or Fisher’s exact test for categorical variables. Between group differences, as well as changes in the intervention group (from baseline), were evaluated for % improvement. Outcomes were evaluated using repeated two-way analysis of variance and assessed differences in intragroup (baseline vs. end of intervention) and intergroup (intervention vs. control). If the findings were significant, we used a Tukey post-hoc test. Clinically meaningful changes in pain were considered when there was a change of three or more points (substantial difference), two to three points in the NRS (moderate difference), or 1 point (minimal difference; Copay et al., 2007; Zonta et al., 2009). A *p* value of 0.05 or less was considered statistically significant.

## Results

### Development of the EE intervention modules

The development of the translated EE intervention involving a review of the existing literature and input from an expert committee and a patient advisory group has been described in detail by Nieves-Vazquez et al. (2022).

### Study subject characteristics

**Figure 1:**
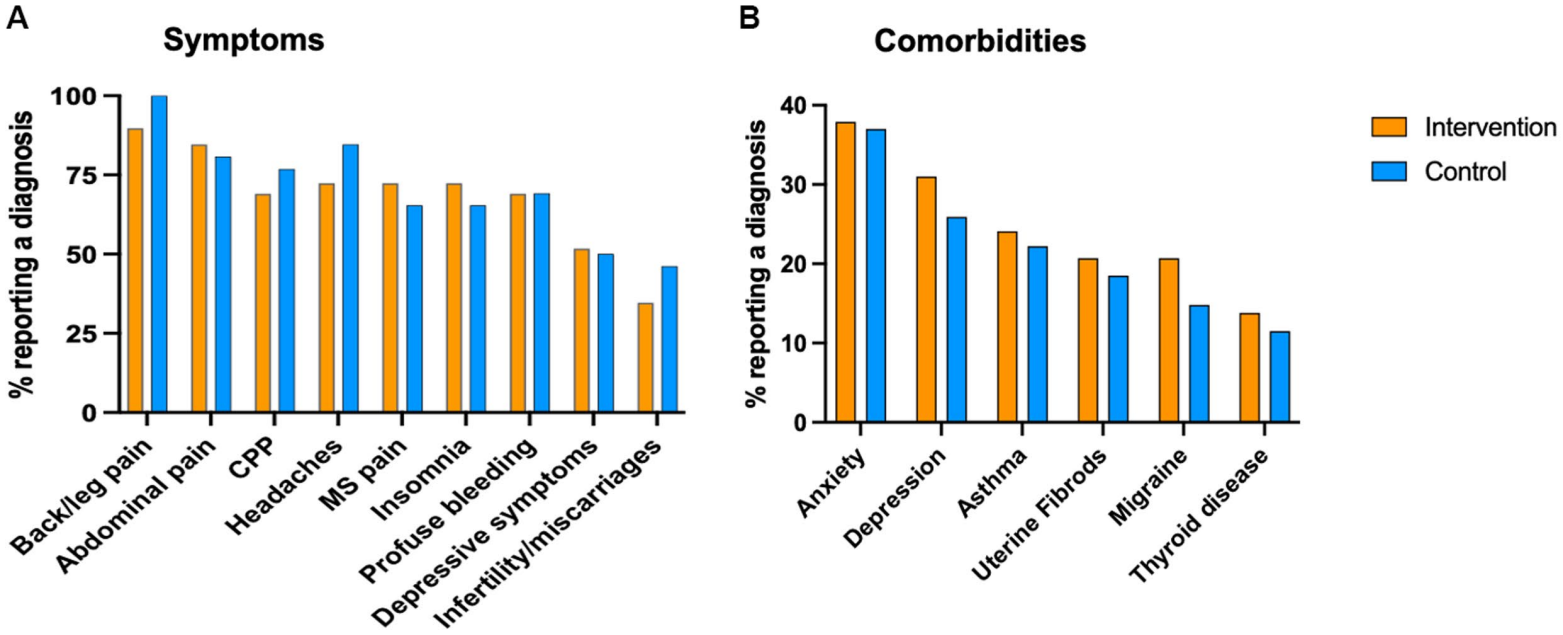
Clinical profile of study participants. Participants completed the EPhect Clinical Questionnaire at baseline. Demographic and clinical variables were self reported. CPP = chronic pelvic pain, MS = musculoesqueletal pain.

The clinical-demographic data from subjects in the study groups are shown in Table 1 and Figure 1. No significant differences were observed between the study groups in any demographic characteristics except education. The proportion of subjects with post-graduate education was significantly higher in the patients in the control vs. intervention group (63% vs. 31%, respectively, *p* = 0.0311). There was a borderline significant difference in age at menarche, and similar rates of endometriosis symptoms and comorbidities, oral contraception use, and pain control medications between the two groups. There were no significant differences between intervention and control groups in the baseline mean levels of current pelvic pain (BPI-NRS: 8.0, SD ± 2.2 vs. 6.4, SD ± 3.3), BPI global impact score (54.14 vs. 51.29), perceived stress (PSS-14: 32.5 SD ± 5.7 vs. 28.0 SD ± 4.4, respectively), nor in Global QoL score (EHP-30: 60.99 vs. 60.10).

**Table 1:** Efficacy of EE for endometriosis.

Study subject characteristics			
	Intervention (*N* = 29)	Control (*N* = 27)	*p* value
Age (Mean in years)	32.7	34.0	0.484
Race			
Mixed	11 (37.9%)	13(48.1%)	
Black	3 (10.3%)	2 (7.4%)	
White	12 (41.3%)	9 (33.3%)	
Other	3 (10.3%)	2 (7.4%)	
Ethnicity			
Hispanic	29 (100%)	27 (100%)	
Anthropometrics			
Height (in.)	64.2	63	0.157
Weight (lbs.)	146.5	139.4	0.767
BMI (Body Mass Index)	23.9	24.8	0.735
Civil status			
Single	16 (55.2%)	15 (55.6%)	>0.999
Committed	13 (44.8%)	12 (44.4%)	
Occupation status			
Study	11 (38.0%)	10 (37.0%)	>0.999
Work	26 (90%)	21 (77.8%)	0.455
Insurance type			
Private	18 (62.1%)	20 (74.1%)	0.766
Public	9 (31.0%)	7 (25.9%)	
None	2 (7.0%)	0 (0%)	
Education			
High School/College	20 (69%)	10 (37%)	**0.031**
Postgraduate	9 (31.0%)	17 (63%)	
Physical activity			
Yes	22 (75.9%)	23 (85.2%)	0.506
Smoking status			
Non-Smoker	27 (93.1%)	27 (100%)	
Alcohol Use			
Yes	15 (51.7%)	12 (44.4%)	0.605
Gynecologic information			
Age at menarche (mean)	12.1	11.2	0.051
Age of onset of pain (mean)	14.3	14.6	0.817
Age diagnosis	24.2	25.5	0.363
Regular cycles	18 (62.1%)	16 (59.3%)	
Pelvic Pain with menses	29 (100%)	24 (88.9%)	
Dyspareunia	26 (89.7%)	21 (77.8%)	0.288
Infertility/miscarriages	9 (31.0%)	12 (46.5%)	0.279
Interventions			
Oral Contraceptive use	11 (38.0%)	11 (40.7%)	
Pain medication use	15 (52.0%)	13 (50%)	
Comorbidities			
Anxiety	11 (37.9%)	10 (37.0%)	
Depression	9 (31.0%)	7 (25.9%)	
Uterine Fibroids	6 (20.7%)	5 (18.5%)	
Asthma	7 (24.1%)	6 (22.2%)	
Migraines	6 (20.7%)	4 (14.8%)	

Sociodemographic data were obtained through the Endometriosis Phenome Project (EPHect) questionnaire, which was sent electronically to all study subjects to be completed at baseline. Bold indicates statistical significance.

### Feasibility and acceptability of the EE intervention

Acceptability of the EE intervention was high, with positive evaluation of the logistics (>70–96% Excellent rates); support groups were rated as Excellent by 91%; 82% would participate regularly; and 96% would recommend the intervention to other patients. Participation rates were 59% for at least a half dose (≥3 interventions or more) and 41% for the full dose. The most common reasons for missing an intervention were pain symptoms and work/family conflicts. Other measures of acceptability and feasibility, including Recruitment, Enrollment, Adherence, Survey completion and Module evaluations, are described in detail by Nieves-Vazquez et al. (2022).

### Pain levels and impact of pain (BPI)

**Figure 2:**
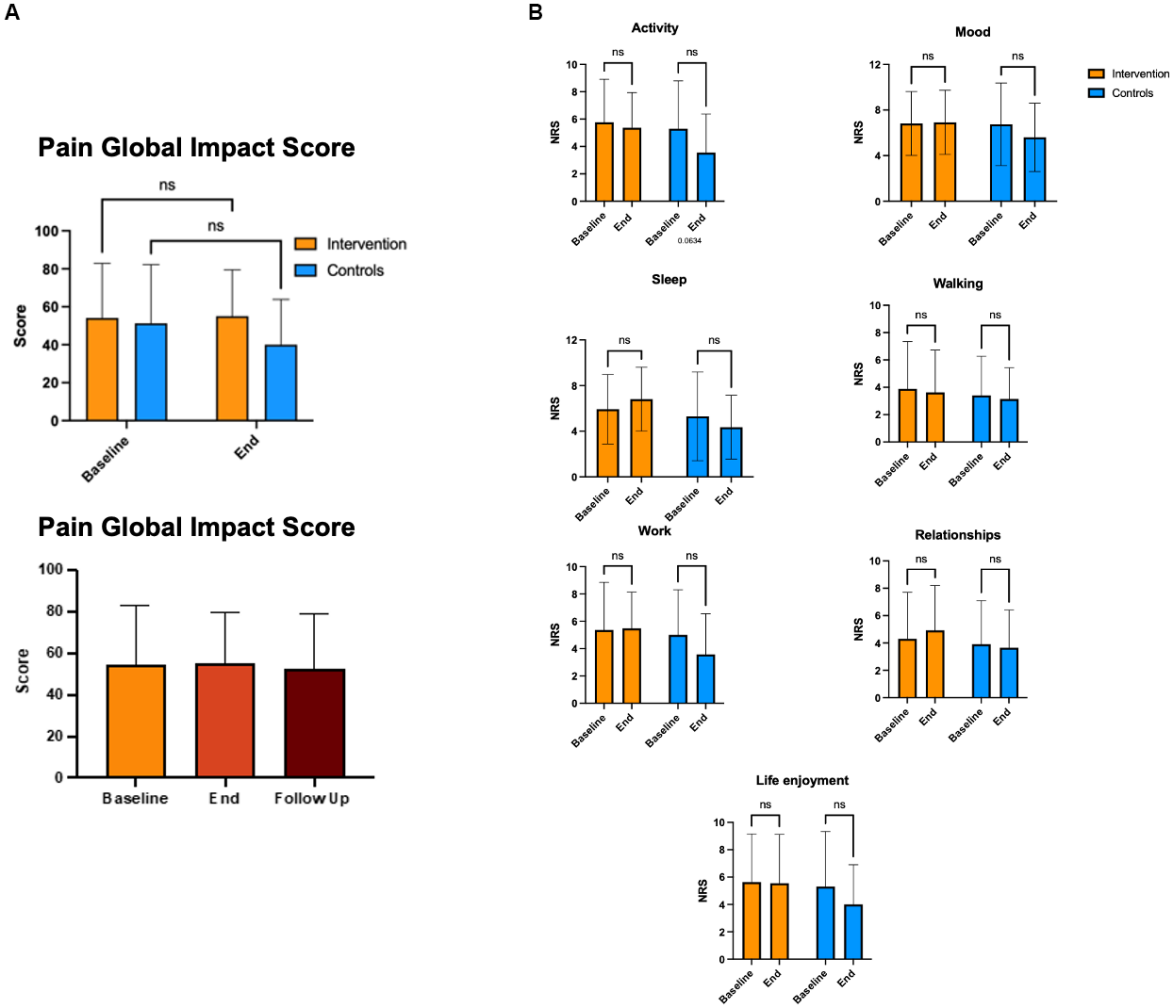
Pain levels and impact of pain (BPI). **(A)** Global impact of endometriosis pain was measured by the BPI survey at baseline, end and follow up (for intervention group). No significant differences were observed between end and baseline in neither group. **(B)** Impact of individual BPI domains were also not significantly different at the end of the intervention in neither group.

**Figure 3:**
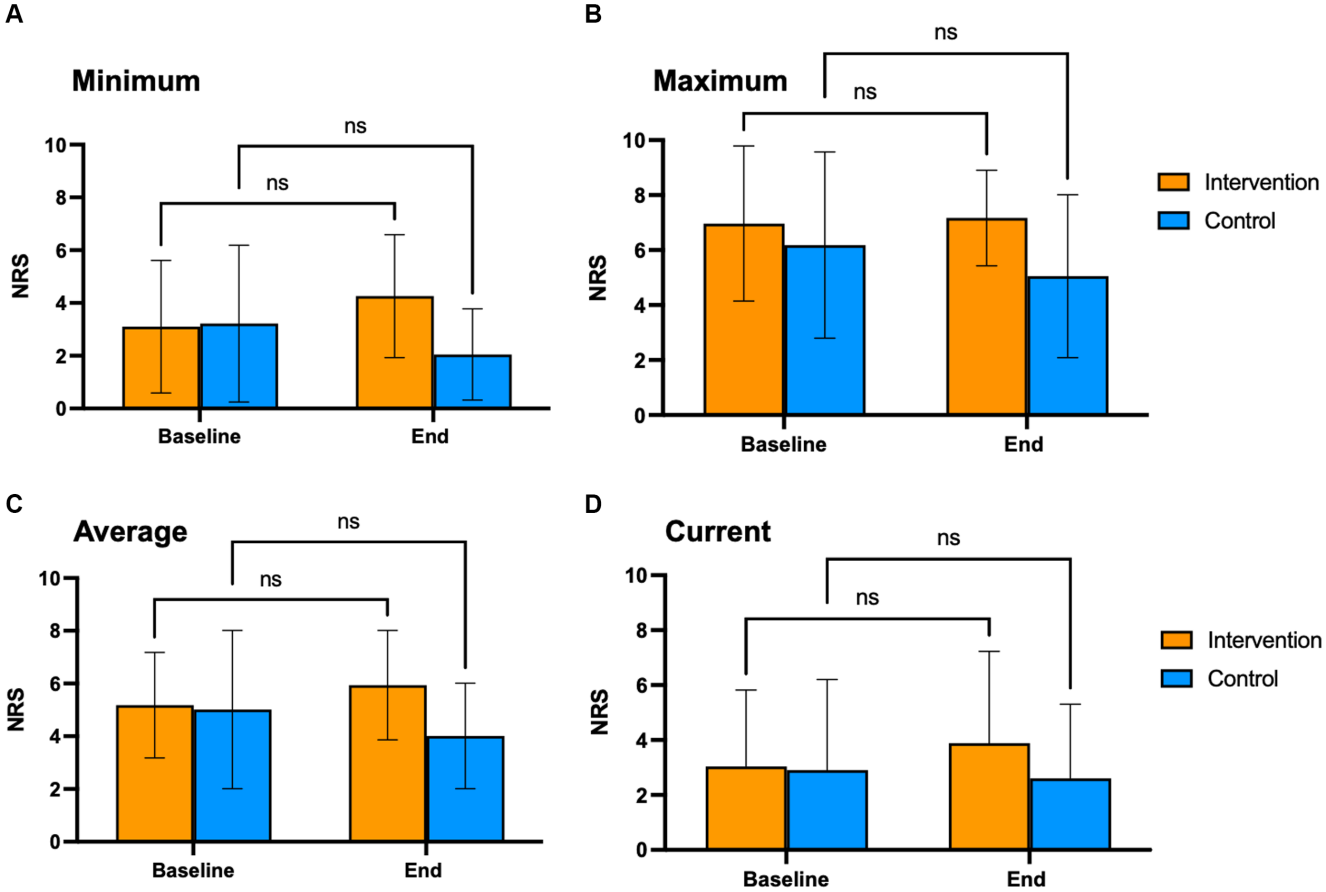
Change in pain levels. Change in pain levels was measured using the BPI survey's NRS scale, that assesses pain intensity at its Minimum **(A)**, Maximum **(B)**, Average **(C)**, and Current **(D)**. NRS scale: 0 = no pain; 10 = worst imaginable pain. This survey was completed at baseline and end of the intervention.

We compared the effects of the EE intervention vs. the control condition using the BPI global impact score, both intragroup (Baseline vs. End of intervention) and intergroup (intervention vs. control; Figure 2A). The intervention and control groups showed similar and not significantly different global pain impact scores at baseline and end of the intervention. No significant changes in the BPI scores were observed in the intervention at the 3-month follow-up. We also compared the effects of the EE intervention vs. the control condition in the different domains of the BPI survey, both intragroup and intergroup. No significant differences were observed in any BPI domains, including activity, mood, walking, relationships, sleep, work, and life enjoyment (Figure 2B). Finally, we analyzed maximum pain levels before and after the intervention and between groups (Figure 3). There were no clinically meaningful changes (moderate to substantial) in the NRS score after the intervention (Copay et al., 2007). Participants in the experimental group reported a mean increase of 1 point in pain at the end of the intervention; at the 3-month follow-up the pain levels were back to baseline. Participants in the control condition showed minimal improvements in pain levels (minimum, maximum, and average; a mean decrease of 1 point in the NRS), but no changes in current pain.

There were no significant differences between groups in the proportion of psychological (57.1% vs. 42.9%) or medical treatment (gynecologic visits, hormonal treatments) during the study (57.1% vs. 42.9%) for the intervention vs. control groups, respectively, that could explain the observed responses regarding pain. At the end of the intervention, 29.6% of subjects in the intervention group were taking hormonal treatments vs. 16.7% of the controls; 55.6% of subjects in the intervention group were taking NSAIDS vs. 70.8% of the controls (n.s.).

### Anxiety symptomatology (GAD-7)

**Figure 4:**
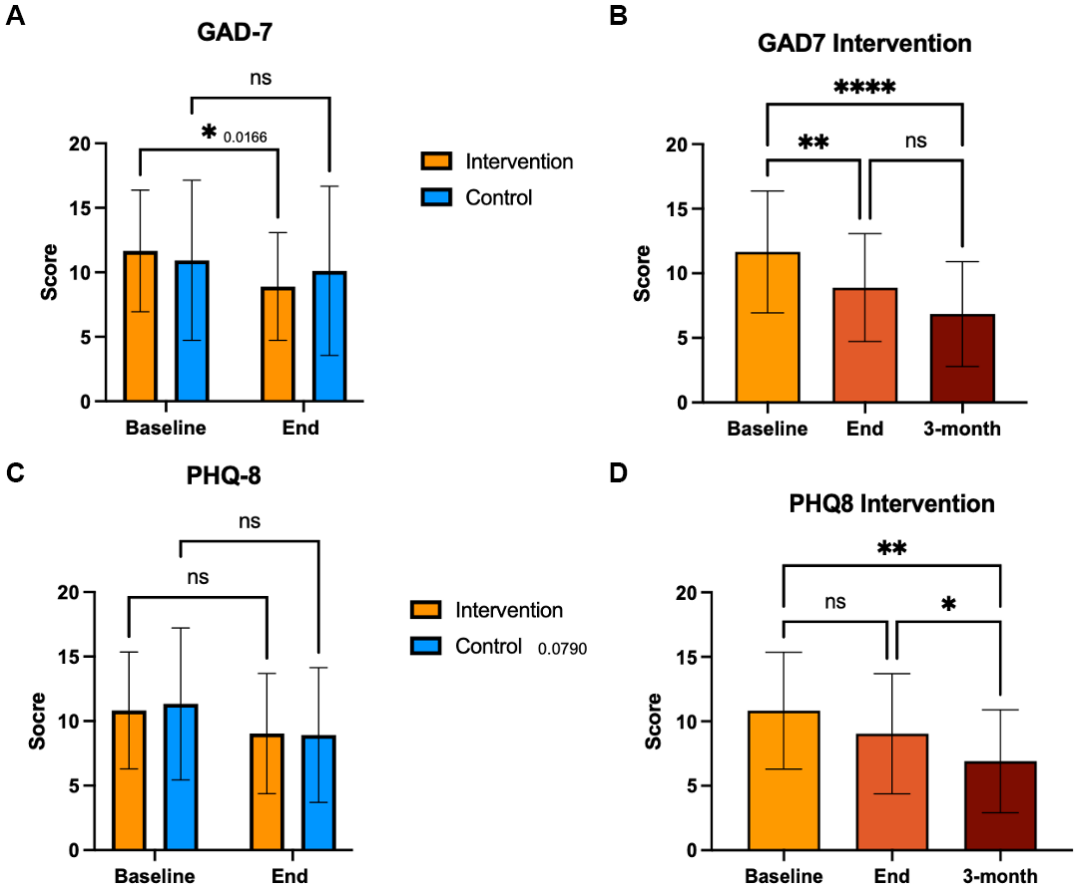
GAD-7 and PHQ-8 scores. Participants completed the GAD-7 and PHQ-8 at baseline end and 3-month follow up. Control group completed these questionnaires at baseline and end of the intervention period. A score of 15 or more is clinically significant.

We compared the effects of the EE intervention vs. the control condition, both intragroup and intergroup, on GAD-7 scores that measure symptoms associated with anxiety (Figure 4A). At baseline, there were no significant differences in mean GAD-7 scores between the intervention (11.7) vs. control group (10.5). We observed a significant difference in GAD-7 scores in the intervention group, which reported lower levels of anxiety at the end of the intervention (*p* = 0.006) and 3-months thereafter (*p* < 0.0001) compared to baseline (Figure 4B). In contrast, the control group showed no significant differences in anxiety levels.

### Depressive symptomatology (PHQ-8)

We compared the effects of the EE intervention vs. the control condition, both intragroup and intergroup, on PHQ-8 scores that measure symptoms associated with depression (Figure 4C). At baseline, the mean PHQ-8 scores were not significantly different between intervention (10.8) and control (11.3) groups. We observed a significant difference in PHQ-8 scores in the intervention group, which reported lower levels of depression at 3-month follow-up compared to both baseline (*p* = 0.006) and to the end of the intervention (*p* = 0.014; Figure 4D). No significant differences were observed for the control group in depressive symptom levels.

**Figure 5:**
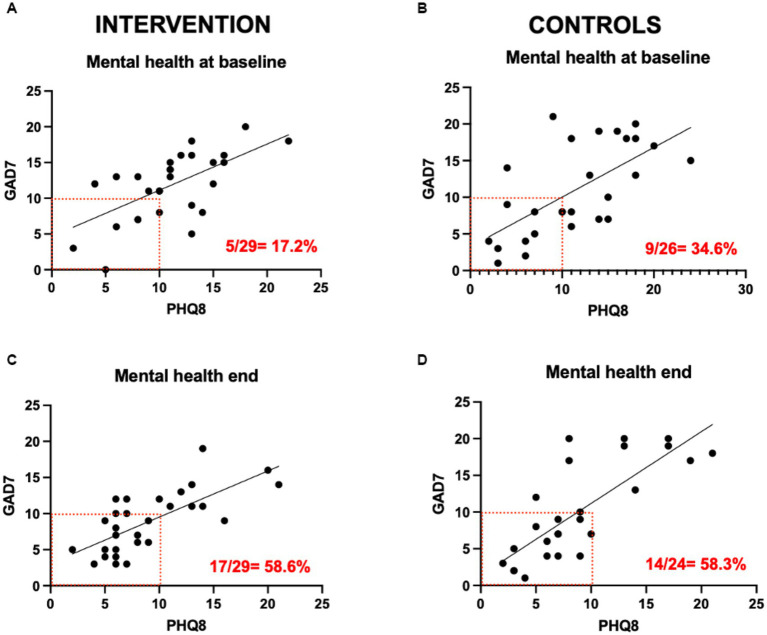
PHQ-8 and GAD-7 scores correlations.

Depressive and anxiety symptoms were strongly correlated in all study subjects at baseline and end of intervention (Figure 5). The proportion of subjects with PHQ-8/GAD-7 scores <10 increased in the intervention group from 17.2 to 58.6% (*p* = 0.003), and in the control group from 34.6 to 58.3% (*p* = 0.17). There was a significant correlation between GAD-7 scores and pain levels (*p* = 0.006) only in the intervention group, which was lost at the end of the intervention (Supplementary Figure 1). There were no significant correlations between PHQ-8 scores and pain levels in either group (Supplementary Figure 2). Endometriosis-related QoL was significantly correlated with GAD-7 and PHQ-8 scores at baseline (*p* = 0.009; *p* = 0.001, respectively) and at the end of the intervention (*p* = 0.008, *p* = 0.012, respectively) in the experimental group, while in the control group, this correlation became significant at the end of the study period (*p* = 0.001, *p* = 0.013, respectively; Supplementary Figures 3, 4).

### Endometriosis-related quality of life (EHP-30)

**Figure 6:**
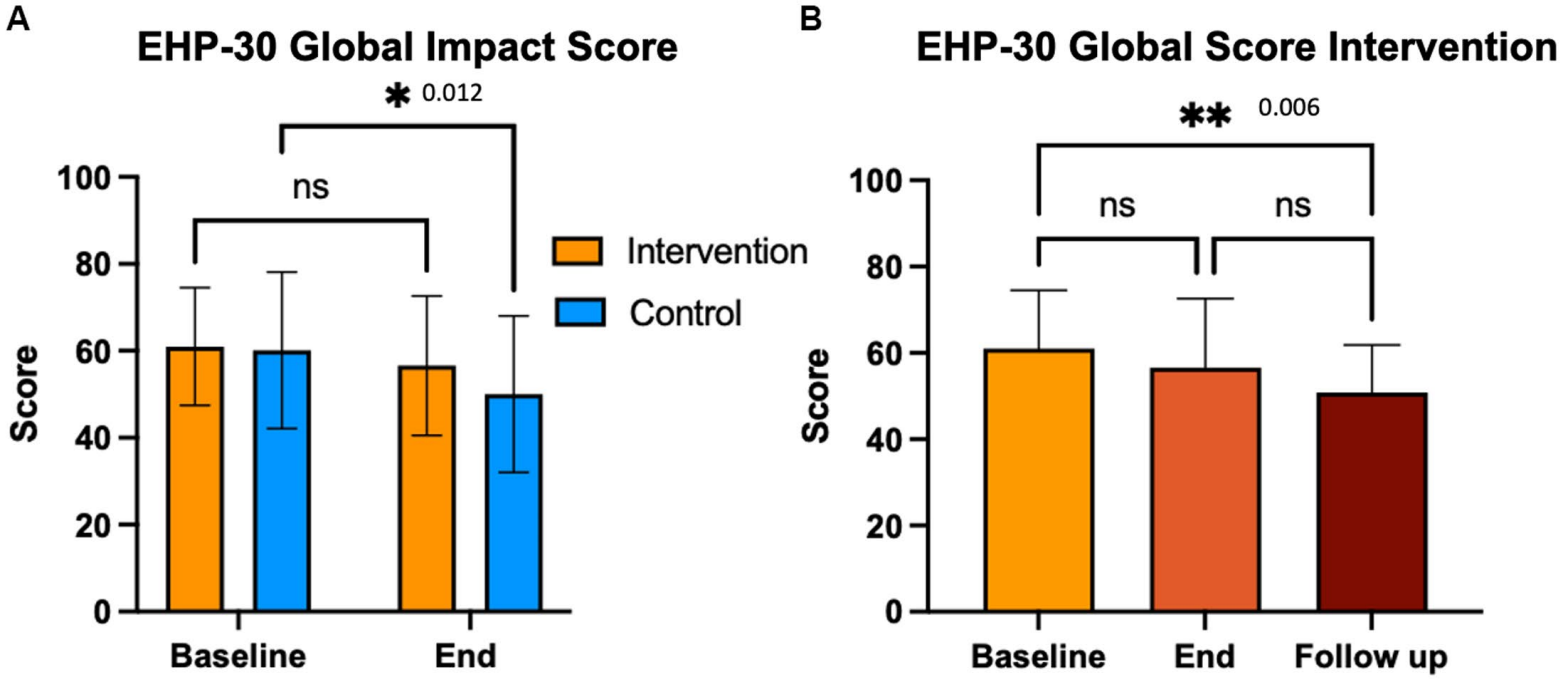
Endometriosis related quality of life (EHP-30). Participants completed the EHP-30 at baseline end and 3-month follow up. Control group completed these questionnaires at baseline and end of the intervention period.

**Figure 7:**
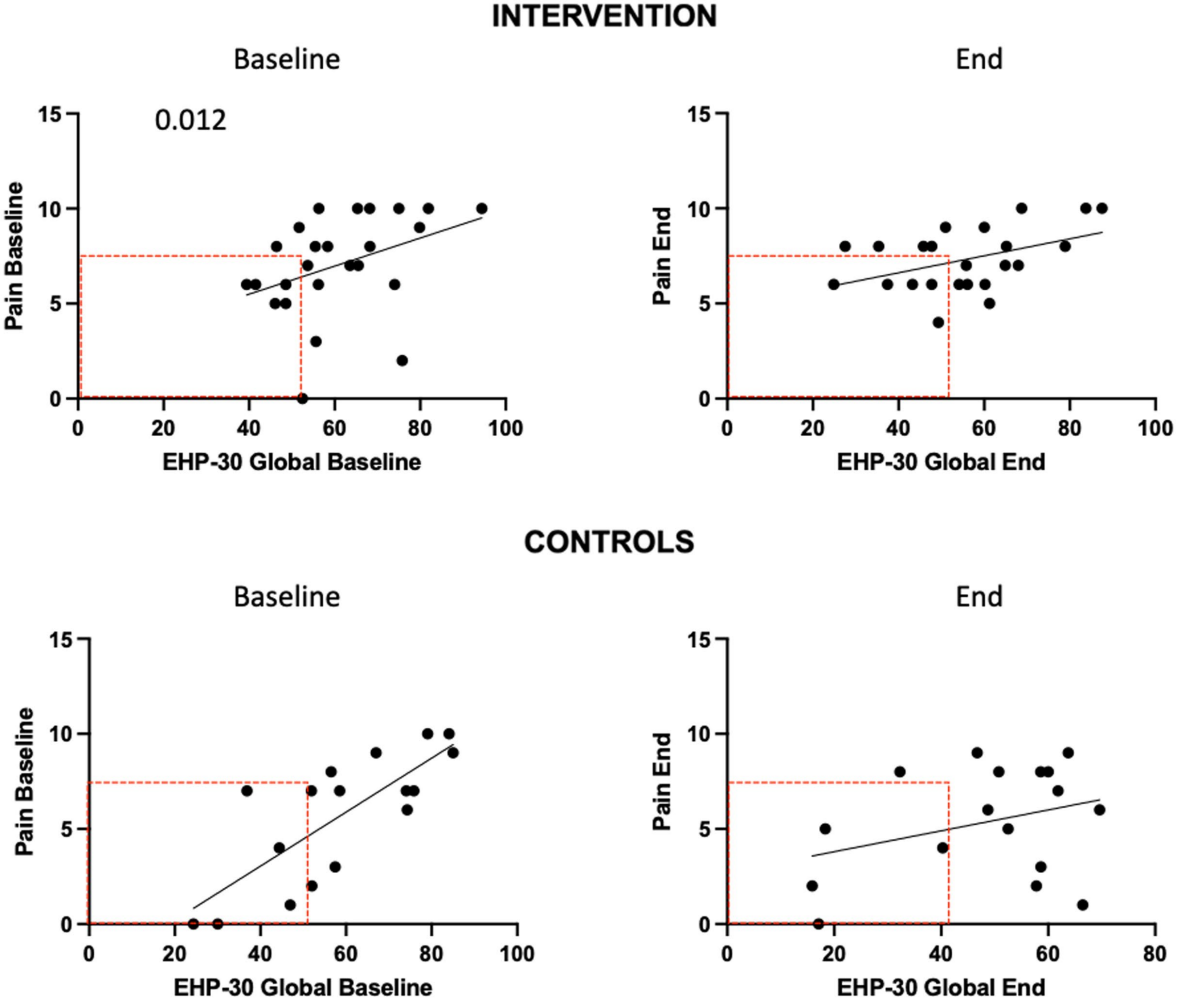
EHP-30 global impact score and pain correlations.

We compared the effects of the EE intervention vs. the control condition, both intragroup and intergroup on QoL (Figure 6). We observed a significant difference in QoL in the control group (*p* = 0.012). Also, a significant decrease in mean EHP-30 global scores was seen at the 3-month follow-up in the intervention group (*p* = 0.006). Of those who improved on QoL global scores, the majority were in the intervention group (61.5% vs. 52.6%, n.s.). QoL scores became significantly correlated with pain levels at the end of the intervention in the experimental group (*p* = 0.034); in the control group the significance between QoL and pain levels was lost at the end of the study (Figure 7).

### Pain catastrophizing (PSC)

**Figure 8:**
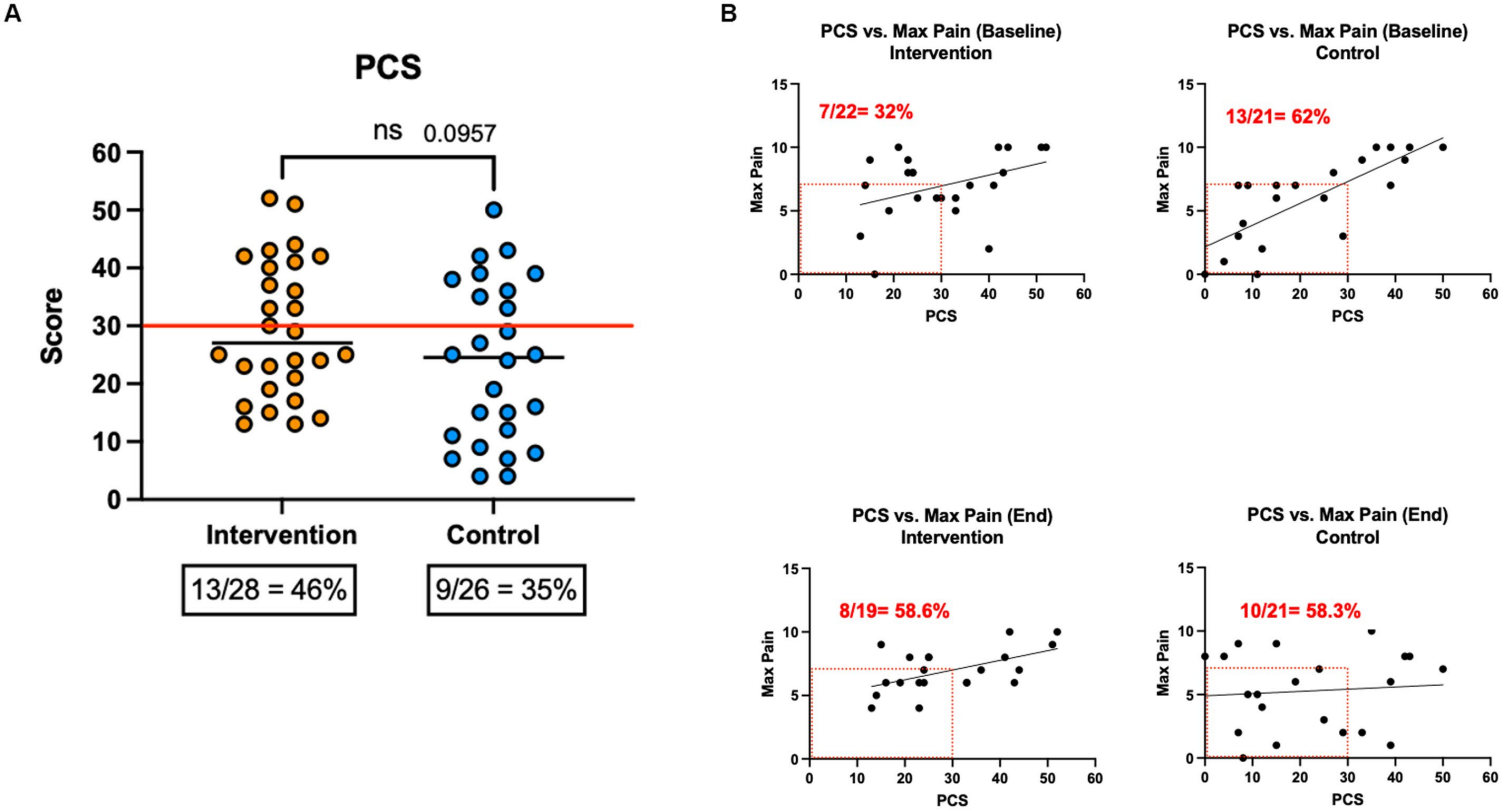
PCS scores and correlations. Participants completed the PCS instrument at baseline. A score of 30 or more is clinically significant.

We assessed whether PCS scores would predict responses to the EE intervention on pain levels and pain impact, measured using BPI. The PCS mean score, measured at baseline, was higher in the intervention vs. control group (29.5 vs. 23.5), but this difference did not reach significance (*p* = 0.096; Figure 8A). The proportion of patients with a score of ≥30, which has clinical relevance, was higher in the intervention condition (46.4% vs. 34.6%, respectively), but this difference was not significant (*p* = 0.42). At baseline, PCS scores were correlated with pain in the intervention (*p* = 0.074) and controls (*p* < 0.0001; Figure 8B). At the end of the study, this association was maintained in the intervention (*p* = 0.011) but not in the control group (n.s). Compared to baseline, the number of patients reporting Low pain (<7)/low PCS scores (<30) increased by the end of the study in the intervention group (32 to 42%) but decreased in the control group (62 to 48%; n.s).

### Perceived stress (PSS-14)

**Figure 9:**
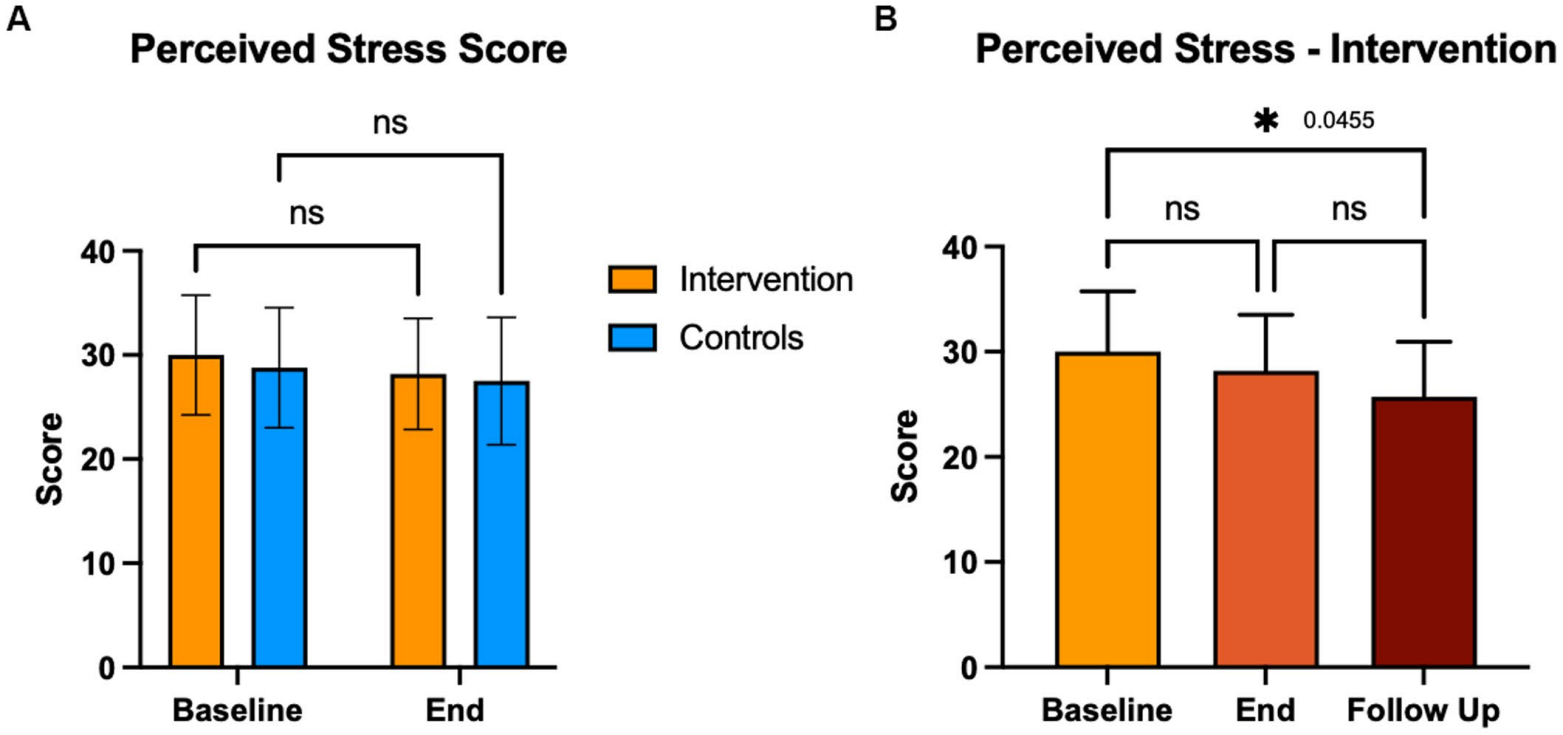
Perceived stress score (PSS-14). Participants completed the PSS-14 at baseline end and 3-month follow up. Control group completed this questionnaire at baseline and end of the intervention period. A score of 38 or more indicates high stress.

We compared the effects of the EE intervention vs. the control condition, both intragroup and intergroup on perceived stress (Figure 9). While we did not observe significant differences in PSS-14 scores immediately after the last intervention, a significant decrease in mean PSS-14 scores was seen at the 3-month follow-up with the intervention (*p* = 0.046). There were no correlations between stress and pain levels at the end of the study in either group.

### Stress reactivity (salivary cortisol)

**Figure 10:**
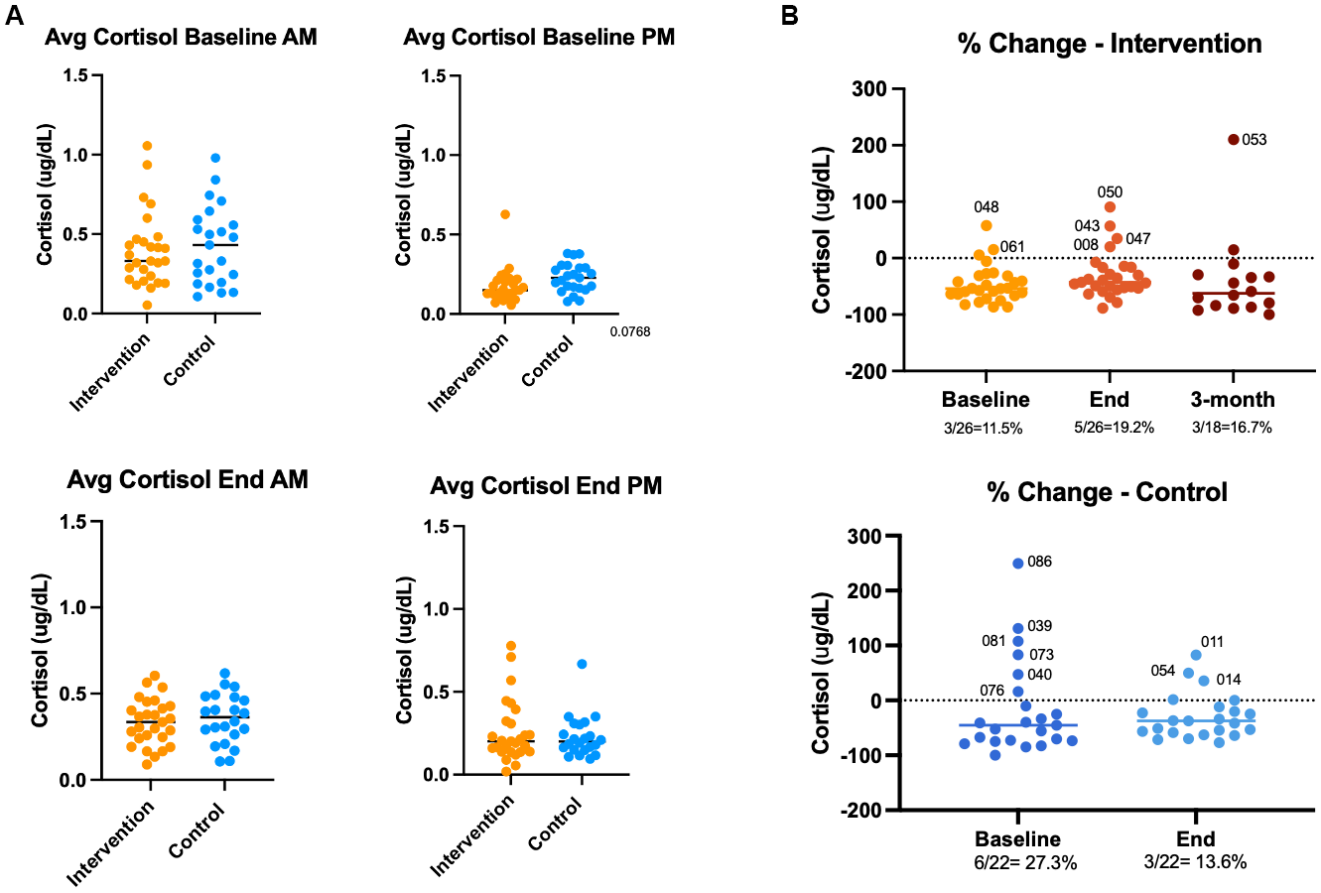
Stress reactivity (salivary cortisol). Participants donated saliva samples at baseline, end and 3-month follow up. Control group donated samples at baseline and end of the intervention period. Samples were collected at ~8 am and at ~12pm by passive drooling. Cortisol levels were analyzed by ELISA, and % PM-AM change were calculated.

We compared the effects of the EE intervention vs. the control condition, both intragroup and intergroup, on the % change in PM vs. AM cortisol levels in saliva (Figure 10A). We did not observe consistent changes indicative of an effect of the EE intervention on cortisol production. Some patients showed cortisol outbursts at the beginning or end of the study period, irrespective of the treatment condition. There were no significant differences in the mean cortisol levels at AM or PM between study groups, at baseline or end of the intervention (Figure 10B).

## Discussion

Based on our previous findings that EE can effectively reduce disease progression and anxiety behaviors in a rat model (Torres-Reverón et al., 2018), our multidisciplinary team translated this paradigm into a multi-modal intervention and tested its efficacy on pain reduction and improved QoL (primary outcomes), stress, inflammation, and mental health (secondary outcomes). First, we determined if the adapted EE intervention would be feasible and acceptable as adjuvant to standard care for endometriosis. As described in the EE intervention was widely accepted by participants who reported high rates of satisfaction with the activities; however, feasibility of the intervention was impacted by the COVID pandemic and patient symptomatology. We report here the results of the first randomized clinical trial to study efficacy of a psychosocial intervention providing an enriched environment consisting of social support, novelty and exposure to open spaces in patients with endometriosis, an inflammatory, painful disease associated with chronic stress.

We evaluated pain levels and impact of pain using the BPI scale that provides a global impact score as well as individual measures of impact in various domains. We did not observe clinically meaningful changes in the NRS score resulting from the intervention nor differences in the BPI global score of pain impact. Due to the dual nature of pain (somatic and emotional) it is challenging to study pain responses in RCTs. There are no known biomarkers that would allow for an objective measure of pain. This leads to high variability in pain as a self-reporting outcome. Future studies could include objective, clinical assessments of pain improvements to validate the self-reporting of changes in pain levels.

Participants of the study could receive standard gynecologic and/or psychological treatments and many did; however, there was no correlation between improvement in global pain impact scores and receiving gynecologic and/or psychological treatments. Our results are in accordance with a vast body of evidence indicating the complexity of treating endometriosis pain. It is well known that many patients with endometriosis are refractory to the available hormonal and analgesic treatments. Medical management of chronic pelvic pain is challenging due to its complex pathophysiology involving biological, social, and psychological factors. Clinically meaningful changes in pain can vary depending on the type of pain and the context, including patient specific factors such as baseline pain level, presence of central sensitization, and expectations that could impact reporting (Raimondo et al., 2023). Hence, it is important to consider a patient’s individual needs and preferences when determining a clinically meaningful change in NRS score.

Because pain catastrophizing can be a factor in non-responses to standard pain treatments, we also analyzed potential associations with this maladaptive coping mechanism. We observed a significant correlation between PCS scores and pain levels indicating that those who reported more catastrophizing did not see improvements in pain. These results are in accord with previous studies showing that pain catastrophizing is a strong predictor of dysmenorrhea severity (Evans et al., 2022). We acknowledge that the EE intervention did not specifically address catastrophizing during the support group meeting, i.e., they were not therapeutically designed to specifically target catastrophizing. Future follow-up studies targeted toward reducing pain catastrophizing, integrating cognitive behavioral therapy (CBT) or acceptance and commitment therapy (ACT), as part of psychosocial adjuvant interventions for endometriosis patients are warranted.

Our results demonstrate that participation in the EE intervention did not cause an improvement in endometriosis related QoL immediately at the end of the study. However, at the 3-month follow-up we observed a significant improvement in the EHP-30 global score in those who participated in the intervention. This may indicate that patients may be more equipped to manage the impact of their condition on QoL by learning and adopting new habits over time. It has been shown that endometriosis symptoms negatively impact QoL at a higher degree than other chronic pain conditions (Centini et al., 2013), thus, the EE intervention shows great promise as a tool to address this critical aspect of wellbeing specifically for patients with endometriosis.

Our pilot RCT shows that participation in at least half of the EE modules led to significantly lower scores of anxiety and depressive symptoms. The beneficial effects of EE on these mood disorders were seen at the end of the intervention and were long-lasting. Anxiety and depression levels were significantly correlated at baseline and at the end of the intervention, meaning that the benefits of EE were impacting both these symptoms concomitantly. Improvements in mental health were independent of pain levels or their impact on work, activity, relationships, and enjoyment, which were not significantly improved. We speculate that exposure to enriched environments can be beneficial for mental health, however, more aggressive standard treatments targeted to pain relief are needed to ensure patients are comprehensively treated.

The mechanisms of improvements in mental health observed in this RCT could be complex and multifactorial. Using an animal model, our team has shown that the “controllability” of stress improved disease presentation (i.e., % lesions developed and size; Appleyard et al., 2015). Furthermore, the EE protocol that incorporates social interaction, open spaces, and cognitive stimulation recapitulated the observed benefits of disease improvements and anxiety amelioration in rodent models of endometriosis, which were correlated with inflammation and HPA axis modulation (Torres-Reverón et al., 2018; Yin et al., 2020). Though we did not observe significant effects of the EE intervention on perceived stress levels at the end of the study, PSS-14 scores decreased significantly at the 3-month follow-up in the intervention group, indicating that an “incubation time” is needed to induce meaningful and long-lasting benefits in the stress response (Stiles et al., 2006; Hofmann et al., 2012). Dysregulations in the HPA axis caused by chronic pain may induce pro-inflammatory mechanisms, impacting disease and mental health (Petrelluzzi et al., 2008; Appleyard et al., 2015; Quiñones et al., 2015), thus, we hypothesized that the beneficial effects of EE in our study could be due to improved stress responses to pain, measured by changes in PM-AM cortisol levels. However, our results are inconclusive in supporting the notion that EE benefits occur *via* direct modulation of the HPA axis. Patients in the intervention and control groups both showed upshots of cortisol output that were intermittent and without a clear trend. It has been previously shown that multiple exposures (including mind–body interventions) can cause acute cortisol changes (Vadiraja et al., 2009; Curtis et al., 2011). Thus, these results must be analyzed with caution. Whether enriched environments could exert an immunomodulatory effect that is long-lasting and beneficial for patients, as has been shown previously, would need to be tested in follow-up RCTs with a larger sample size (Shields et al., 2020). In addition, brain imaging or other systemic biomarkers could shed light on whether activation of brain–body–brain pathways is at play (Beissner et al., 2018)

Notably, we observed significant improvements in the control group that had a wait-list condition, meaning that subjects knew that they would eventually participate in the intervention. When analyzing the results using scatter plots, we noted that two control patients showed consistent drastic improvements in several domains (over 2 SD from the mean) of pain and QoL that caused a decrease in the mean global score (data not shown). Follow-up interviews of these patients identified potential modulating effects of pain reporting such as psychological treatment prompted by participation in the study, recent diagnosis and pharmacological treatment coinciding with participation in the study, and the hopeful effect of knowing that they will be part of the 2nd intervention group. Some verbatims from the interviews are:

“I felt hope that there would be light at the end of the tunnel.”

“I was hopeful because I felt that I was going to obtain tools and new knowledge about the condition.”

“I felt that I would have an escape, meet other women going through the same; I felt I could contribute with a “grain of sand” (to do one’s bit) no matter which study group I was in.”

Qualitative analysis of patient interviews indicates that there was a positive effect of knowing that they will eventually receive the intervention. As shown in other studies (Morone et al., 2009), expectant control groups could benefit from the attention received and hope from knowing they were on a wait-list and contributing to research. Other limitations of our pilot study include the high attrition rate and the resulting small sample size. Finally, the study design did not include a 3-month follow-up for the controls, as it involved substantial time and effort of a large team to complete the house visits for collecting samples and surveys, which precluded inter-group comparison of long-term effects. Considering the challenges of conducting RCTs with extensive time-involvement, especially during a pandemic, we believe the results of this pilot study contribute to the field by providing evidence-based support for a psychosocial intervention with significant, long-lasting changes in anxiety symptomatology that participants considered highly valuable and impactful.

The beneficial effects of integrative medicine strategies involving psychological interventions and stress management for pain, inflammation and mood disorders are well-established. Among the most studied modalities are yoga, psychotherapy, exercise, and mindfulness/meditation (Beck and Beamesderfer, 1974; Spitzer et al., 2006; Perera et al., 2017). Chronic pelvic pain, a common symptom of endometriosis, causes substantial biopsychosocial impact that makes its clinical management challenging. This results in suboptimal or short-term responses to the available therapeutic alternatives for this complex symptom, including analgesia, hormonal therapies, and surgery. Despite the obvious need to incorporate mind–body alternatives for the management of endometriosis, there are only three published RCTs involving yoga, systemic autoregulation therapy (SART; consisting of psychotherapy plus Chinese medicine), and progressive muscular relaxation (Evans et al., 2019). All three RCTs showed significant improvements in pain, anxiety and depressive symptoms (Zhao et al., 2012; Meissner et al., 2016; Goncalves et al., 2017). Follow-up mechanistic studies using qualitative analysis of interviews and brain imaging showed that these strategies provided increased awareness due to the integration of body, mind and breath, increased psychosocial support, and reduced connectivity in brain areas related to the HPA axis. In addition, there have been three single-arm trials (physical therapy plus psychotherapy, mindfulness and psychotherapy), and one retrospective cohort study (on SART), providing some evidence for a benefit in pain levels, mood and fatigue (reviewed by Evans et al. (2019)). As a result of the limited evidence-based studies published, patients remain at a loss with respect to alternative options for managing symptoms when pharmacological/surgical therapies fail.

Our study addressed a critical gap in endometriosis research by evaluating the efficacy of an integrative, psychosocial intervention based on the enriched environments paradigm through a rigorous randomized clinical trial that showed significant and long-lasting improvements in anxiety and depressive symptoms, despite no impact on pain (Hansen et al., 2023). Together, these RCTs document the benefits of utilizing *integrative, multi-level* approaches for managing endometriosis biopsychosocial impacts, and support integrating alternative symptom management options to standard therapies that often provide only partial, short-term relief.

## Conclusion

In conclusion, the enriched environment behavioral paradigm, encompassing psychological (group support), behavioral (novelty), and stress reduction (open environment) interventions, was highly acceptable to patients and demonstrated significant and long-lasting improvements in anxiety and depressive symptoms, stress and QoL. As an integral part of a patient-centered clinical management plan, this integrative, multi-level approach should be considered an adjuvant to standard care, aiming to address the complex biopsychosocial impacts of endometriosis and provide comprehensive relief to patients. Further research with larger sample sizes and longer follow-up periods is needed to elucidate the mechanisms of action and validate the benefits of enriched environments in endometriosis management.

## Data availability statement

The raw data supporting the conclusions of this article will be made available by the authors, without undue reservation.

## Ethics statement

The studies involving humans were approved by Ponce Research Institute Institutional Review Board (IRB). The studies were conducted in accordance with the local legislation and institutional requirements. The participants provided their written informed consent to participate in this study.

## Author contributions

All authors made substantial contributions to the study, drafted the manuscript or revised it critically, and approved the version to be published. All authors agree to be accountable for all aspects of the work, its accuracy and integrity. GD and DR-S contributed equally to this work. IF, AT-R, CA, EC, and FS-S: conception and design of the work. GD, DR-S, VL-R, CN-V, and BB-C: data acquisition. IF, AT-R, BB-C, GD, DR-S, CN-V, CA, and EC: data analysis. IF, AT-R, CA, EC, and BB-C: interpretation of data. IF, AT-R, CA, CN-V, GD, DR-S, VL-R, EC, and BB-C: Writing and revising manuscript.

## References

[ref1] Appleyard C. B. Cruz M. L. Hernández S. Thompson K. J. Bayona M. Flores I. (2015). Stress management affects outcomes in the pathophysiology of an endometriosis model. Reprod. Sci. 22, 431–441. doi: 10.1177/1933719114542022, PMID: 25015902 PMC4812689

[ref2] Appleyard C. B. Flores I. Torres-Reveron A. (2020). The link between stress and endometriosis: from animal models to the clinical scenario. Reprod. Sci. 27, 1675–1686. doi: 10.1007/s43032-020-00205-7, PMID: 32542543 PMC9262098

[ref3] Barros W. David M. Souza A. Silva M. Matos R. (2019). Can the effects of environmental enrichment modulate BDNF expression in hippocampal plasticity? A systematic review of animal studies. Synapse 73:e22103. doi: 10.1002/syn.22103, PMID: 31056812

[ref4] Beck A. T. Beamesderfer A. (1974). Assessment of depression: the depression inventory. Mod. Probl. Pharmacopsychiatry 7, 151–169. doi: 10.1159/000395074 4412100

[ref5] Beissner F. Preibisch C. Schweizer-Arau A. Popovici R. M. Meissner K. (2018). Psychotherapy with somatosensory stimulation for endometriosis-associated pain: the role of the anterior Hippocampus. Biol. Psychiatry 84, 734–742. doi: 10.1016/j.biopsych.2017.01.006, PMID: 28258747

[ref6] Benaroya-Milshtein N. Hollander N. Apter A. Kukulansky T. Raz N. Wilf A.. (2004). Environmental enrichment in mice decreases anxiety, attenuates stress responses and enhances natural killer cell activity. Eur. J. Neurosci. 20, 1341–1347. doi: 10.1111/j.1460-9568.2004.03587.x, PMID: 15341605

[ref7] Brasil D. L. Montagna E. Trevisan C. M. la Rosa V. L. Laganà A. S. Barbosa C. P.. (2020). Psychological stress levels in women with endometriosis: systematic review and meta-analysis of observational studies. Minerva Med. 111, 90–102. doi: 10.23736/S0026-4806.19.06350-X, PMID: 31755674

[ref8] Casalechi M. Vieira-Lopes M. Quessada M. P. Arão T. C. Reis F. M. (2021). Endometriosis and related pelvic pain: association with stress, anxiety and depressive symptoms. Minerva Obstet. Gynecol. 73, 283–289. doi: 10.23736/S2724-606X.21.04704-3, PMID: 34008383

[ref9] Centini G. L. L. Dores D. Pianigiani L. Iannone P. Luisi S. Petraglia Z. E. (2013). Chronic pelvic pain and quality of life in women with and without endometriosis. J. Endo. Pelvic Pain Dis. 5, 27–33. doi: 10.5301/JE.5000148

[ref10] Copay A. G. Subach B. R. Glassman S. D. Polly D. W. Jr. Schuler T. C. (2007). Understanding the minimum clinically important difference: a review of concepts and methods. Spine J. 7, 541–546. doi: 10.1016/j.spinee.2007.01.008 17448732

[ref11] Cuevas M. Cruz M. L. Ramirez A. E. Flores I. Thompson K. J. Bayona M.. (2018). Stress during development of experimental endometriosis influences nerve growth and disease progression. Reprod. Sci. 25, 347–357. doi: 10.1177/1933719117737846, PMID: 29108503 PMC6343219

[ref12] Cuevas M. Flores I. Thompson K. J. Ramos-Ortolaza D. L. Torres-Reveron A. Appleyard C. B. (2012). Stress exacerbates endometriosis manifestations and inflammatory parameters in an animal model. Reprod. Sci. 19, 851–862. doi: 10.1177/1933719112438443, PMID: 22527982 PMC4046310

[ref13] Curtis K. Osadchuk A. Katz J. (2011). An eight-week yoga intervention is associated with improvements in pain, psychological functioning and mindfulness, and changes in cortisol levels in women with fibromyalgia. J. Pain Res. 4, 189–201. doi: 10.2147/JPR.S22761, PMID: 21887116 PMC3160832

[ref14] Dandi E. Kalamari A. Touloumi O. Lagoudaki R. Nousiopoulou E. Simeonidou C.. (2018). Beneficial effects of environmental enrichment on behavior, stress reactivity and synaptophysin/BDNF expression in hippocampus following early life stress. Int. J. Dev. Neurosci. 67, 19–32. doi: 10.1016/j.ijdevneu.2018.03.003, PMID: 29545098

[ref15] de la Tremblaye P. B. Cheng J. P. Bondi C. O. Kline A. E. (2019). Environmental enrichment, alone or in combination with various pharmacotherapies, confers marked benefits after traumatic brain injury. Neuropharmacology 145, 13–24. doi: 10.1016/j.neuropharm.2018.02.032 29499273

[ref16] de Sousa Fernandes M. S. Santos G. C. J. Filgueira T. O. Gomes D. A. Barbosa E. A. S. dos Santos T. M.. (2022). Cytokines and immune cells profile in different tissues of rodents induced by environmental enrichment: systematic review. Int. J. Mol. Sci. 23:11986. doi: 10.3390/ijms231911986 36233282 PMC9570198

[ref17] Evans S. Dowding C. Olive L. Payne L. A. Druitt M. Seidman L. C.. (2022). Pain catastrophizing, but not mental health or social support, is associated with menstrual pain severity in women with dysmenorrhea: a cross-sectional survey. Psychol. Health Med. 27, 1410–1420. doi: 10.1080/13548506.2021.1948581 34190659

[ref18] Evans S. Fernandez S. Olive L. Payne L. A. Mikocka-Walus A. (2019). Psychological and mind-body interventions for endometriosis: a systematic review. J. Psychosom. Res. 124:109756. doi: 10.1016/j.jpsychores.2019.109756, PMID: 31443810

[ref19] Flores-Ramos M. Yoldi-Negrete M. Guiza-Zayas R. Ramírez-Rodríguez G. B. Montes-Castrejón A. Fresán A. (2022). An Indicator of environmental enrichment to measure physical, social and cognitive activities in human daily life. BMC Psychiatry 22:295. doi: 10.1186/s12888-022-03952-w 35468768 PMC9040238

[ref20] Fournier A. P. Baudron E. Wagnon I. Aubert P. Vivien D. Neunlist M.. (2020). Environmental enrichment alleviates the deleterious effects of stress in experimental autoimmune encephalomyelitis. Mult. Scler J. Exp. Transl. Clin. 6:2055217320959806. doi: 10.1177/2055217320959806, PMID: 33101703 PMC7550951

[ref21] Fourquet J. Báez L. Figueroa M. Iriarte R. I. Flores I. (2011). Quantification of the impact of endometriosis symptoms on health-related quality of life and work productivity. Fertil. Steril. 96, 107–112. doi: 10.1016/j.fertnstert.2011.04.095, PMID: 21621771 PMC3129383

[ref22] Fourquet J. Gao X. Zavala D. Orengo J. C. Abac S. Ruiz A.. (2010). Patients' report on how endometriosis affects health, work, and daily life. Fertil. Steril. 93, 2424–2428. doi: 10.1016/j.fertnstert.2009.09.017, PMID: 19926084 PMC2860000

[ref23] Gabriel A. F. Paoletti G. Seta D. D. Panelli R. Marcus M. A. E. Farabollini F.. (2010). Enriched environment and the recovery from inflammatory pain: social versus physical aspects and their interaction. Behav. Brain Res. 208, 90–95. doi: 10.1016/j.bbr.2009.11.015, PMID: 19914294

[ref24] Goncalves A. V. Barros N. F. Bahamondes L. (2017). The practice of hatha yoga for the treatment of pain associated with endometriosis. J. Altern. Complement. Med. 23, 45–52. doi: 10.1089/acm.2015.0343, PMID: 27869485

[ref25] Gonçalves L. V. Herlinger A. L. Ferreira T. A. A. Coitinho J. B. Pires R. G. W. Martins-Silva C. (2018). Environmental enrichment cognitive neuroprotection in an experimental model of cerebral ischemia: biochemical and molecular aspects. Behav. Brain Res. 348, 171–183. doi: 10.1016/j.bbr.2018.04.023, PMID: 29684474

[ref26] Hansen K. E. Brandsborg B. Kesmodel U. S. Forman A. Kold M. Pristed R.. (2023). Psychological interventions improve quality of life despite persistent pain in endometriosis: results of a 3-armed randomized controlled trial. Qual. Life Res. 32, 1727–1744. doi: 10.1007/s11136-023-03346-9, PMID: 36797461 PMC10172241

[ref27] Hernandez S. Cruz M. L. Seguinot I. I. Torres-Reveron A. Appleyard C. B. (2017). Impact of psychological stress on pain perception in an animal model of endometriosis. Reprod. Sci. 24, 1371–1381. doi: 10.1177/1933719116687655, PMID: 28093054 PMC5933089

[ref28] Hofmann S. G. Asnaani A. Vonk I. J. J. Sawyer A. T. Fang A. (2012). The efficacy of cognitive behavioral therapy: a review of meta-analyses. Cognit. Ther. Res. 36, 427–440. doi: 10.1007/s10608-012-9476-1, PMID: 23459093 PMC3584580

[ref29] Jenkinson C. Kennedy S. Jones G. (2008). Evaluation of the American version of the 30-item endometriosis health profile (EHP-30). Qual. Life Res. 17, 1147–1152. doi: 10.1007/s11136-008-9403-9 18846435

[ref30] Jones G. Jenkinson C. Kennedy S. (2004). Evaluating the responsiveness of the endometriosis health profile questionnaire: the EHP-30. Qual. Life Res. 13, 705–713. doi: 10.1023/B:QURE.0000021316.79349.af, PMID: 15130032

[ref31] Kimura L. F. Novaes L. S. Picolo G. Munhoz C. D. Cheung C. W. Camarini R. (2022). How environmental enrichment balances out neuroinflammation in chronic pain and comorbid depression and anxiety disorders. Br. J. Pharmacol. 179, 1640–1660. doi: 10.1111/bph.15584, PMID: 34076891

[ref32] Kristiansen F. L. Olesen A. E. Brock C. Gazerani P. Petrini L. Mogil J. S.. (2014). The role of pain catastrophizing in experimental pain perception. Pain Pract. 14, E136–E145. doi: 10.1111/papr.12150, PMID: 24219590

[ref33] Liew A. K. Y. Teo C. H. Soga T. (2022). The molecular effects of environmental enrichment on Alzheimer's disease. Mol. Neurobiol. 59, 7095–7118. doi: 10.1007/s12035-022-03016-w, PMID: 36083518 PMC9616781

[ref34] Lima A. P. Moura M. D. Rosa e Silva A. A. M. (2006). Prolactin and cortisol levels in women with endometriosis. Braz. J. Med. Biol. Res. 39, 1121–1127. doi: 10.1590/S0100-879X2006000800015, PMID: 16906287

[ref35] Meissner K. Schweizer-Arau A. Limmer A. Preibisch C. Popovici R. M. Lange I.. (2016). Psychotherapy with somatosensory stimulation for endometriosis-associated pain: a randomized controlled trial. Obstet. Gynecol. 128, 1134–1142. doi: 10.1097/AOG.0000000000001691, PMID: 27741200

[ref36] Miettinen T. Kautiainen H. Mäntyselkä P. Linton S. J. Kalso E. (2019). Pain interference type and level guide the assessment process in chronic pain: categorizing pain patients entering tertiary pain treatment with the brief pain inventory. PLoS One 14:e0221437. doi: 10.1371/journal.pone.0221437, PMID: 31430355 PMC6701883

[ref37] Missmer S. A. Tu F. F. Agarwal S. K. Chapron C. Soliman A. M. Chiuve S.. (2021). Impact of endometriosis on life-course potential: a narrative review. Int J Gen Med 14, 9–25. doi: 10.2147/IJGM.S261139, PMID: 33442286 PMC7800443

[ref38] Morone N. E. Rollman B. L. Moore C. G. Li Q. Weiner D. K. (2009). A mind-body program for older adults with chronic low back pain: results of a pilot study. Pain Med. 10, 1395–1407. doi: 10.1111/j.1526-4637.2009.00746.x, PMID: 20021599 PMC2849802

[ref39] Neves L.T. Paz L.V. Wieck A. Mestriner R.G. de Miranda Monteiro V.A.C. Xavier L.L., Environmental enrichment in stroke research: an update. Transl. Stroke Res. (2023). doi: 10.1007/s12975-023-01132-w, PMID: [Epub ahead of print]. 36717476

[ref40] Nieves-Vazquez C. I. Detrés-Marquéz A. C. Torres-Reverón A. Appleyard C. B. Llorens-De Jesús A. P. Resto I. N.. (2022). Feasibility and acceptability of an adapted environmental enrichment intervention for endometriosis: a pilot study. Front. Glob. Womens Health 3:1058559. doi: 10.3389/fgwh.2022.1058559, PMID: 36683601 PMC9846621

[ref41] Perera M. J. Brintz C. E. Birnbaum-Weitzman O. Penedo F. J. Gallo L. C. Gonzalez P.. (2017). Factor structure of the perceived stress Scale-10 (PSS) across English and Spanish language responders in the HCHS/SOL sociocultural ancillary study. Psychol. Assess. 29, 320–328. doi: 10.1037/pas0000336, PMID: 27280744 PMC5148735

[ref42] Petrelluzzi K. F. Garcia M. C. Petta C. A. Grassi-Kassisse D. M. Spadari-Bratfisch R. C. (2008). Salivary cortisol concentrations, stress and quality of life in women with endometriosis and chronic pelvic pain. Stress 11, 390–397. doi: 10.1080/10253890701840610, PMID: 18800310

[ref43] Petrini L. Arendt-Nielsen L. (2020). Understanding pain catastrophizing: putting pieces together. Front. Psychol. 11:603420. doi: 10.3389/fpsyg.2020.603420, PMID: 33391121 PMC7772183

[ref44] Qin H. Reid I. Gorelik A. Ng L. (2021). Environmental enrichment for stroke and other non-progressive brain injury. Cochrane Database Syst. Rev. 11:CD011879. doi: 10.1002/14651858.CD011879.pub2, PMID: 34811724 PMC8609277

[ref45] Queen N. J. Hassan Q. N. 2nd Cao L. (2020). Improvements to healthspan through environmental enrichment and lifestyle interventions: where are we now? Front. Neurosci. 14:605. doi: 10.3389/fnins.2020.00605, PMID: 32655354 PMC7325954

[ref46] Quiñones M. Urrutia R. Torres-Reverón A. Vincent K. Flores I. (2015). Anxiety, coping skills and hypothalamus-pituitary-adrenal (HPA) axis in patients with endometriosis. J Reprod Biol Health 3:2. doi: 10.7243/2054-0841-3-2, PMID: 26900480 PMC4755521

[ref47] Raimondo D. Raffone A. Renzulli F. Sanna G. Raspollini A. Bertoldo L.. (2023). Prevalence and risk factors of central sensitization in women with endometriosis. J. Minim. Invasive Gynecol. 30, 73–80.e1 e1. doi: 10.1016/j.jmig.2022.10.007, PMID: 36441085

[ref48] Rojas-Carvajal M. Sequeira-Cordero A. Brenes J. C. (2022). The environmental enrichment model revisited: a translatable paradigm to study the stress of our modern lifestyle. Eur. J. Neurosci. 55, 2359–2392. doi: 10.1111/ejn.15160, PMID: 33638921

[ref49] Rosbergen I. C. Grimley R. S. Hayward K. S. Brauer S. G. (2019). The impact of environmental enrichment in an acute stroke unit on how and when patients undertake activities. Clin. Rehabil. 33, 784–795. doi: 10.1177/0269215518820087, PMID: 30582368

[ref50] Scarola S. J. Perdomo Trejo J. R. Granger M. E. Gerecke K. M. Bardi M. (2019). Immunomodulatory effects of stress and environmental enrichment in long-evans rats (*Rattus norvegicus*). Comp. Med. 69, 35–47. doi: 10.30802/AALAS-CM-18-000025, PMID: 30728094 PMC6382044

[ref51] Seong H. H. Park J. M. Kim Y. J. (2018). Antidepressive effects of environmental enrichment in chronic stress-induced depression in rats. Biol. Res. Nurs. 20, 40–48. doi: 10.1177/1099800417730400, PMID: 28931312

[ref52] Shields G. S. Spahr C. M. Slavich G. M. (2020). Psychosocial interventions and immune system function: a systematic review and meta-analysis of randomized clinical trials. JAMA Psychiatry 77, 1031–1043. doi: 10.1001/jamapsychiatry.2020.0431, PMID: 32492090 PMC7272116

[ref53] Simpson J. Kelly J. P. (2011). The impact of environmental enrichment in laboratory rats--behavioural and neurochemical aspects. Behav. Brain Res. 222, 246–264. doi: 10.1016/j.bbr.2011.04.002, PMID: 21504762

[ref54] Singhal G. Morgan J. Jawahar M. C. Corrigan F. Jaehne E. J. Toben C.. (2019). The effects of short-term and long-term environmental enrichment on locomotion, mood-like behavior, cognition and hippocampal gene expression. Behav. Brain Res. 368:111917. doi: 10.1016/j.bbr.2019.111917, PMID: 31004685

[ref55] Singhal G. Jaehne E. J. Corrigan F. Baune B. T. (2014). Cellular and molecular mechanisms of immunomodulation in the brain through environmental enrichment. Front. Cell. Neurosci. 8:97. doi: 10.3389/fncel.2014.00097, PMID: 24772064 PMC3982075

[ref56] Spitzer R. L. Kroenke K. Williams J. B. W. Löwe B. (2006). A brief measure for assessing generalized anxiety disorder: the GAD-7. Arch. Intern. Med. 166, 1092–1097. doi: 10.1001/archinte.166.10.1092 16717171

[ref57] Stiles W. B. Barkham M. Twigg E. Mellor-Clark J. Cooper M. (2006). Effectiveness of cognitive-behavioural, person-centred and psychodynamic therapies as practised in UK National Health Service settings. Psychol. Med. 36, 555–566. doi: 10.1017/S0033291706007136, PMID: 16476185

[ref58] Sullivan M.L., The pain catastrophizing scale user manual. (2009). Montreal, Quebec: McGill University.

[ref59] Sztainberg Y. Kuperman Y. Tsoory M. Lebow M. Chen A. (2010). The anxiolytic effect of environmental enrichment is mediated via amygdalar CRF receptor type 1. Mol. Psychiatry 15, 905–917. doi: 10.1038/mp.2009.151, PMID: 20084060

[ref60] Tapias V. Moschonas E. H. Bondi C. O. Vozzella V. J. Cooper I. N. Cheng J. P.. (2022). Environmental enrichment improves traumatic brain injury-induced behavioral phenotype and associated neurodegenerative process. Exp. Neurol. 357:114204. doi: 10.1016/j.expneurol.2022.114204, PMID: 35973617

[ref61] Torres-Reverón A. Rivera L. L. Flores I. Appleyard C. B. (2018). Environmental manipulations as an effective alternative treatment to reduce endometriosis progression. Reprod. Sci. 25, 1336–1348. doi: 10.1177/1933719117741374, PMID: 29137551 PMC6346300

[ref62] Vadiraja H. S. Raghavendra R. M. Nagarathna R. Nagendra H. R. Rekha M. Vanitha N.. (2009). Effects of a yoga program on cortisol rhythm and mood states in early breast cancer patients undergoing adjuvant radiotherapy: a randomized controlled trial. Integr. Cancer Ther. 8, 37–46. doi: 10.1177/1534735409331456, PMID: 19190034

[ref63] Vannuccini S. Lazzeri L. Orlandini C. Morgante G. Bifulco G. Fagiolini A.. (2018). Mental health, pain symptoms and systemic comorbidities in women with endometriosis: a cross-sectional study. J. Psychosom. Obstet. Gynaecol. 39, 315–320. doi: 10.1080/0167482X.2017.1386171, PMID: 29027829

[ref64] Vitonis A. F. Vincent K. Rahmioglu N. Fassbender A. Buck Louis G. M. Hummelshoj L.. (2014). World endometriosis research foundation endometriosis phenome and biobanking harmonization project: II. Clinical and covariate phenotype data collection in endometriosis research. Fertil. Steril. 102, 1223–1232. doi: 10.1016/j.fertnstert.2014.07.1244, PMID: 25256930 PMC4252538

[ref65] Wang X. M. Zhang G. F. Jia M. Xie Z. M. Yang J. J. Shen J. C.. (2019). Environmental enrichment improves pain sensitivity, depression-like phenotype, and memory deficit in mice with neuropathic pain: role of NPAS4. Psychopharmacology 236, 1999–2014. doi: 10.1007/s00213-019-5187-6, PMID: 30798405

[ref66] Yin B. Jiang H. Liu X. Guo S. W. (2020). Enriched environment decelerates the development of endometriosis in mouse. Reprod. Sci. 27, 1423–1435. doi: 10.1007/s43032-019-00117-1, PMID: 32318984

[ref67] Zhao L. Wu H. Zhou X. Wang Q. Zhu W. Chen J. (2012). Effects of progressive muscular relaxation training on anxiety, depression and quality of life of endometriosis patients under gonadotrophin-releasing hormone agonist therapy. Eur. J. Obstet. Gynecol. Reprod. Biol. 162, 211–215. doi: 10.1016/j.ejogrb.2012.02.029, PMID: 22455972

[ref68] Zonta S. De Martino M. Dionigi P. (2009). Requirements for applying a case-control study model and clinical significance of changes in the visual analogue scale score in abdominal pain. Surg. Endosc. 23, 227–228. doi: 10.1007/s00464-008-0175-7, PMID: 18855058

