# Supplementary material for: Efficacy of an environmental enrichment intervention for endometriosis: a pilot study
Source: Front Psychol. 2023 Oct 10;14:1225790. doi: 10.3389/fpsyg.2023.1225790 (PMC10598732; doi:10.3389/fpsyg.2023.1225790)
Supplement: Supplementary file 2 [file Image_2.pdf]

Supplemental Figure 2

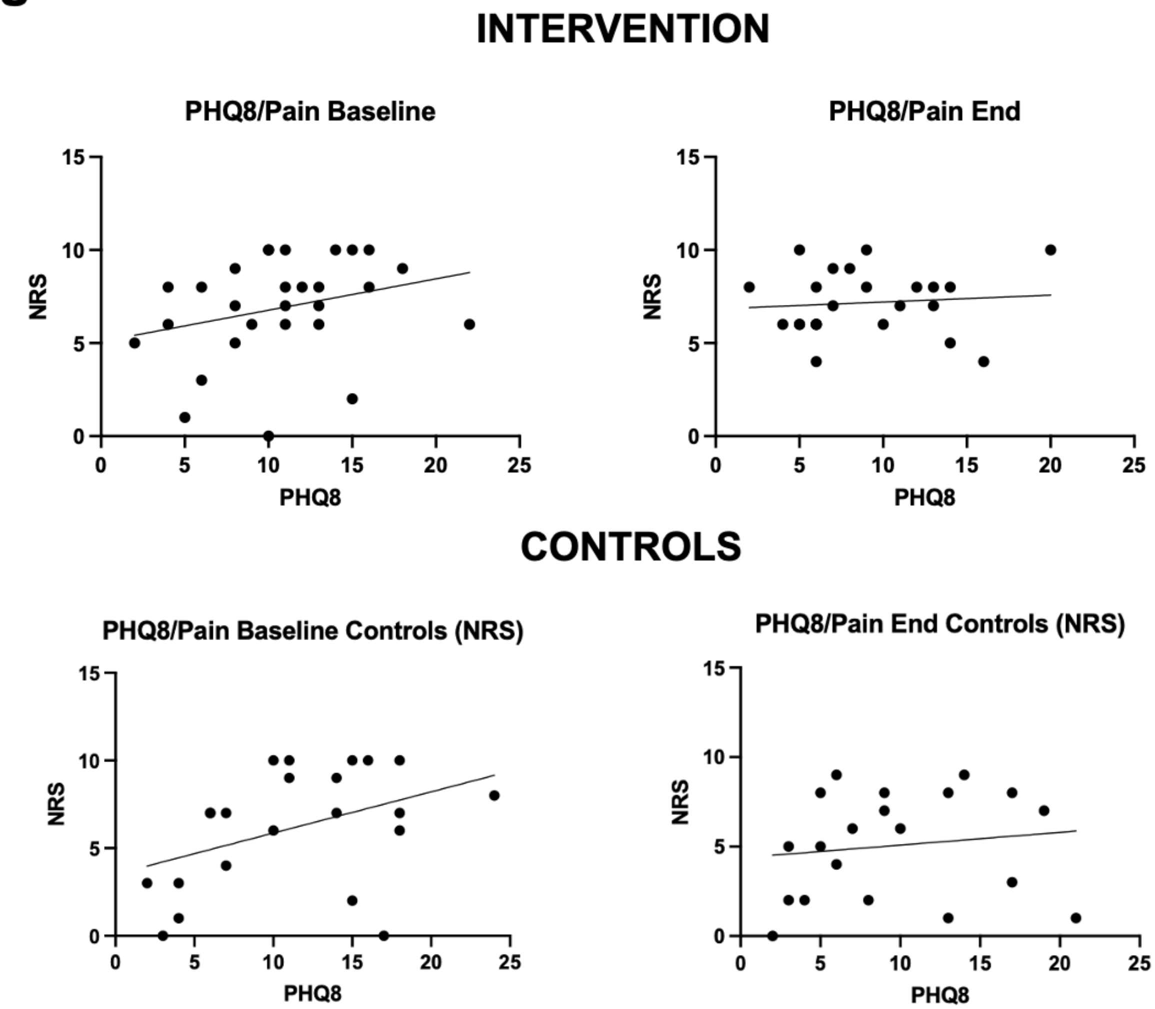

Figure SF2: Correlations between NRS (pain) and depression (PHQ8) among participants in the intervention and control groups
