# Supplementary figures and images for: Efficacy of an environmental enrichment intervention for endometriosis: a pilot study
Source: Front Psychol. 2023 Oct 10;14:1225790. doi: 10.3389/fpsyg.2023.1225790 (PMC10598732; doi:10.3389/fpsyg.2023.1225790)

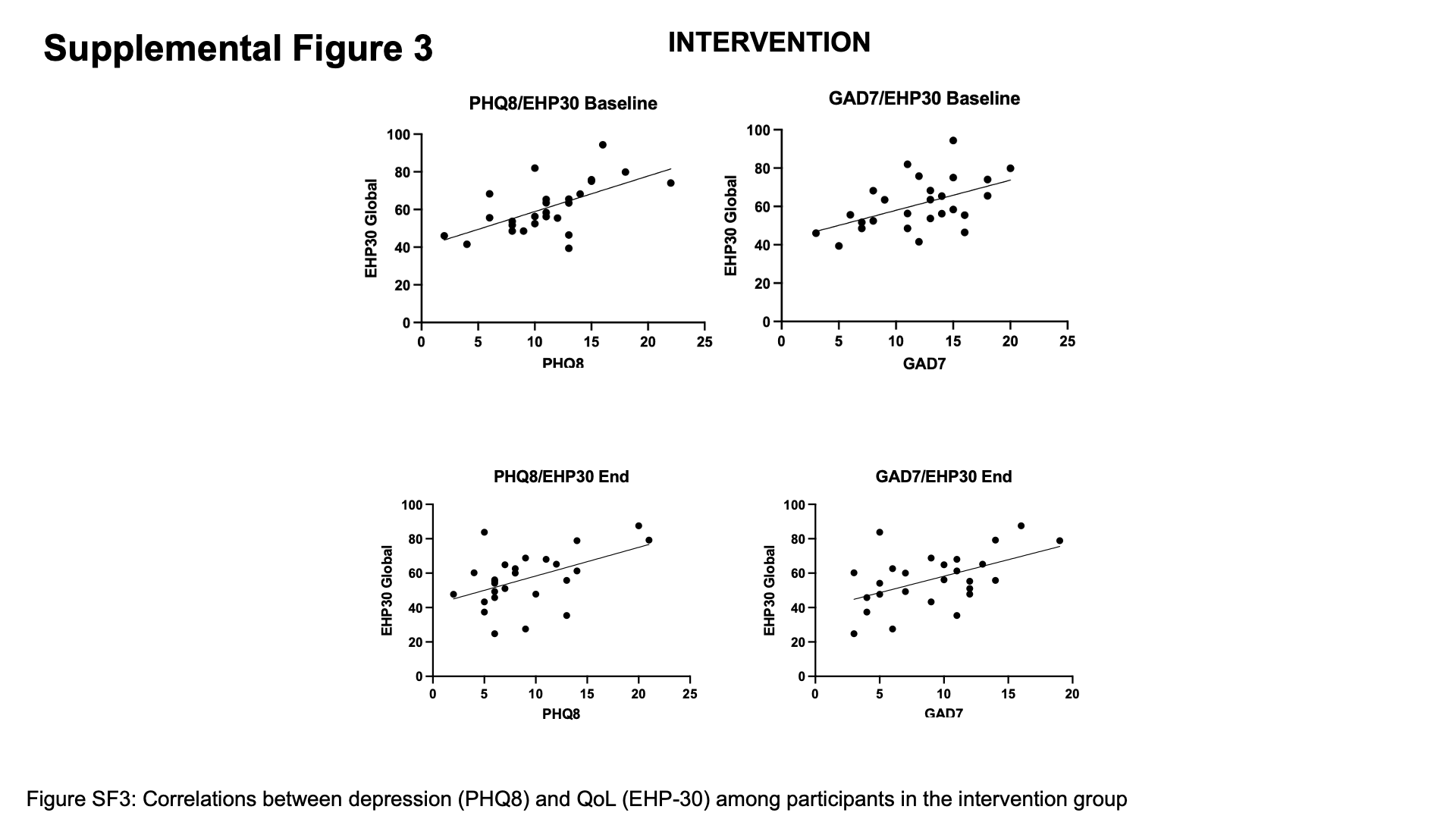

Supplement: Supplementary file 3 [file Image_3.tiff]
