# Supplementary material for: Efficacy of an environmental enrichment intervention for endometriosis: a pilot study
Source: Front Psychol. 2023 Oct 10;14:1225790. doi: 10.3389/fpsyg.2023.1225790 (PMC10598732; doi:10.3389/fpsyg.2023.1225790)
Supplement: Supplementary file 4 [file Image_4.pdf]

Supplemental Figure 4

CONTROLS

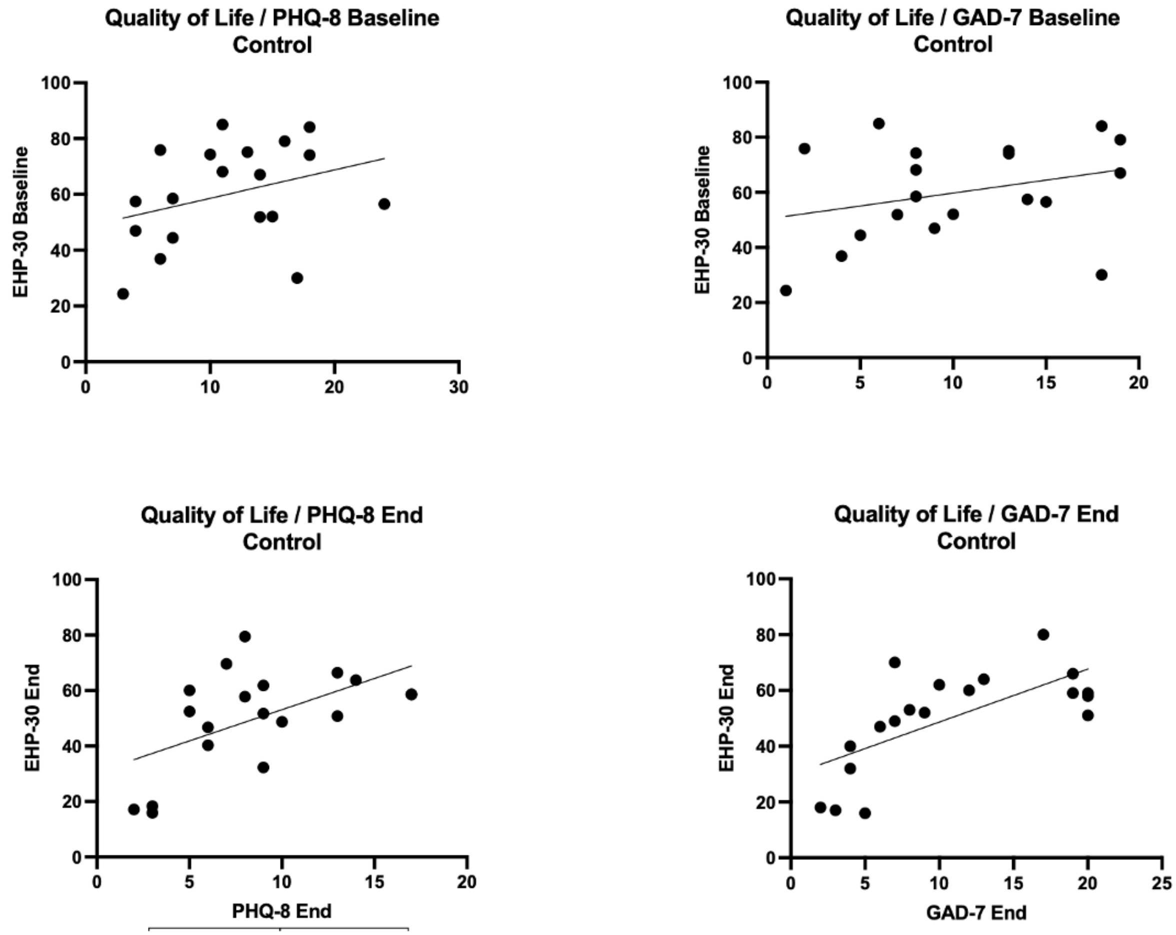

Figure SF4: Correlations between depression (PHQ8) and QoL (EHP-30) among participants in the control group
